# Systematic review of machine learning-based radiomics approach for predicting microsatellite instability status in colorectal cancer

**DOI:** 10.1007/s11547-023-01593-x

**Published:** 2023-01-17

**Authors:** Qiang Wang, Jianhua Xu, Anrong Wang, Yi Chen, Tian Wang, Danyu Chen, Jiaxing Zhang, Torkel B. Brismar

**Affiliations:** 1 Division of Medical Imaging and Technology, Department of Clinical Science, Intervention and Technology (CLINTEC), Karolinska Institutet, Stockholm, Sweden; 2 Department of Radiology, Karolinska University Hospital Huddinge, Room 601, Novum PI 6, Hiss F, Hälsovägen 7, 141 86 Huddinge, Stockholm, Sweden; 3 Department of General Surgery, Songshan Hospital, Chongqing, China; 4 Department of Vascular Surgery, The First Affiliated Hospital of Chongqing Medical University, Chongqing, China; 5 Department of Interventional Therapy, People’s Hospital of Dianjiang County, Chongqing, China; 6 Department of Oncology-Pathology, Karolinska Institutet, Stockholm, Sweden; 7 Department of Gastroenterology, Chongqing General Hospital, Chongqing, China; 8 Department of Gastroenterology and Hepatology, Sun Yat-Sen Memorial Hospital, Sun Yat-Sen University, Guangzhou, China; 9 Department of Pharmacy, Guizhou Provincial People’s Hospital, Guiyang, China

**Keywords:** Radiomics, Microsatellite instability, Colorectal neoplasms, Machine learning, Systematic review as topic

## Abstract

This study aimed to systematically summarize the performance of the machine learning-based radiomics models in the prediction of microsatellite instability (MSI) in patients with colorectal cancer (CRC). It was conducted according to the preferred reporting items for a systematic review and meta-analysis of diagnostic test accuracy studies (PRISMA-DTA) guideline and was registered at the PROSPERO website with an identifier CRD42022295787. Systematic literature searching was conducted in databases of PubMed, Embase, Web of Science, and Cochrane Library up to November 10, 2022. Research which applied radiomics analysis on preoperative CT/MRI/PET-CT images for predicting the MSI status in CRC patients with no history of anti-tumor therapies was eligible. The radiomics quality score (RQS) and Quality Assessment of Diagnostic Accuracy Studies 2 (QUADAS-2) were applied to evaluate the research quality (full score 100%). Twelve studies with 4,320 patients were included. All studies were retrospective, and only four had an external validation cohort. The median incidence of MSI was 19% (range 8–34%). The area under the receiver operator curve of the models ranged from 0.78 to 0.96 (median 0.83) in the external validation cohort. The median sensitivity was 0.76 (range 0.32–1.00), and the median specificity was 0.87 (range 0.69–1.00). The median RQS score was 38% (range 14–50%), and half of the studies showed high risk in patient selection as evaluated by QUADAS-2. In conclusion, while radiomics based on pretreatment imaging modalities had a high performance in the prediction of MSI status in CRC, so far it does not appear to be ready for clinical use due to insufficient methodological quality.

**Supplementary Information:**

The online version contains supplementary material available at 10.1007/s11547-023-01593-x.

## Introduction

Colorectal cancer (CRC) ranks as the third most common malignant tumor and the second leading cause of cancer-related death globally [1]. Microsatellite instability (MSI) is a well-established cancer hallmark that is defined as the generalized instability of the short, non-sense, repeat DNA sequences (i.e., microsatellites) due to a deficient repair system of the DNA mismatches at replication. About 13–15% of CRC patients have tumors with MSI [2, 3]. It occurs more often in older patients, in right-sided locations, and has a lower pathological stage, representing a distinct CRC subtype [4].

Clinical decision-making can benefit from the information on pre-treatment MSI status for patients with CRC. Patients with MSI often have better outcomes and are less likely to have lymph node spread and metastasis [2, 5]. Besides, patients with CRC MSI generally do not benefit from preoperative 5-fluorouracil-based adjuvant therapy [6–8]. Under this context, MSI testing has been recommended for all patients with stage II rectal patients by the National Comprehensive Cancer Network practice guidelines since 2016 [9]. Furthermore, MSI status can also serve as a predictor for the response to immunotherapy [10, 11]. Previous studies have shown that MSI CRC patients are sensitive to immune checkpoint inhibitors due to the high expression level of mutant neoantigens [12, 13]. Therefore, the European Society for Medical Oncology recommends MSI evaluation before immunotherapy [14] and the US Food and Drug Administration has approved MSI as an indication for cancer immunotherapy [15].

At present, MSI status is mainly evaluated through immunohistochemistry or polymerase chain reaction on specimens obtained by colonoscopy biopsy or surgical resection [2]. However, information about mismatch repair protein express level obtained postoperatively exerts little influence on the pretreatment planning, and the limited samples obtained via biopsy may not thoroughly reflect the intra-tumoral heterogeneity [16]. In some cases, a false negative result may occur (2.1–5.9%) [17]. In addition, biopsy and surgery are also invasive procedures, leaving the patients at risk of procedure-related complications and are not practical for repeated monitoring [18]. A non-invasive, reliable, and cost-effective approach to identifying the MSI status would be of great value.

Imaging modalities, such as computed tomography (CT), magnetic resonance imaging (MRI), and positron emission tomography/CT (PET/CT), are commonly used for the detection, characterization, and staging of CRC. The subtle information underlying these images may reflect the genetic/molecular alterations of CRC, such as MSI [19]. By using modern computing techniques, the imaging information can be mined and converted to quantitative high-dimension data, and the latter can be further exploited for the construction of prediction models via machine learning algorithms—this technique has been coined as “radiomics” [19–22].In recent years, plenty of studies using the radiomics approach for CRC MSI status prediction have emerged [22]. However, the reported prediction accuracy and efficacy of these radiomics models vary and the overall performance remains unknown. To date, there is not any research summarizing current evidence about radiomics methods for MSI status prediction in CRC patients. Such summaries are of clinical importance for evidence-based patient management. This systematic review was therefore aimed to summarize the current evidence and to provide a summary of the predictive performance of the radiomics models in the diagnosis of MSI in CRC. In addition, the research and reporting quality of these studies were also evaluated.

## Materials and methods

This study was conducted according to the Preferred Reporting Items for a Systematic Review and Meta-analysis of Diagnostic Test Accuracy Studies (PRISMA-DTA) guideline [23], and the checklist can be found in Supplementary file 1. The research protocol has been registered at the PROSPERO website (https://www.crd.york.ac.uk/prospero/) under registration No. CRD42022295787.

### Literature search

A systematic literature search was performed to detect any potentially relevant publications at four public databases: PubMed, Embase, Web of Science, and Cochrane Library with key terms of “colorectal cancer (CRC)/colon cancer/rectal cancer/colorectal liver metastases (CRLM)”, “microsatellite instability (MSI)/mismatch repair deficient (dMMR)” and “radiomics/texture analysis/radiogenomics/imaging biomarker”, their synonyms, and their Medical Subject Headings terms (detailed search queries are provided in Supplementary file 2). The literature search was first conducted on April 15 2022 and last updated on November 10 2022.

### Study selection

Studies meeting the following inclusion and exclusion criteria were regarded as eligible and included in this research. Inclusion criteria: 1) retrospective or prospective design; 2) patients with CRC confirmed by postoperative histopathological examination and no history of anti-tumor therapies (i.e., neoadjuvant chemotherapy or radiation therapy) before imaging examinations; 3) radiomics features extracted from the entire volume of the lesion at CT, MRI or PET/CT examinations and used as a single predictor or one of the variables in a prediction model; 4) MSI status was evaluated on the surgical specimens; 5) publications in English. Exclusion criteria: 1) publications in the form of review, conference abstract, corrigendum, book chapter, or study protocol; 2) research outcomes not involving MSI; 3) deep learning research; 4) sample size of less than 50 patients.

Two researchers ('Q.W' and 'J.X', with 7 and 2 years of experience in preparing and updating systematic reviews, respectively) conducted study selection independently, first by screening the title and abstract and then by reading the full text of the potentially eligible studies. The disagreement was solved by discussion or consultancy with a senior researcher ('T.B.B'). In addition, review and cited references in the included articles were manually identified to detect any eligible research.

### Data extraction

**Fig. 1:**
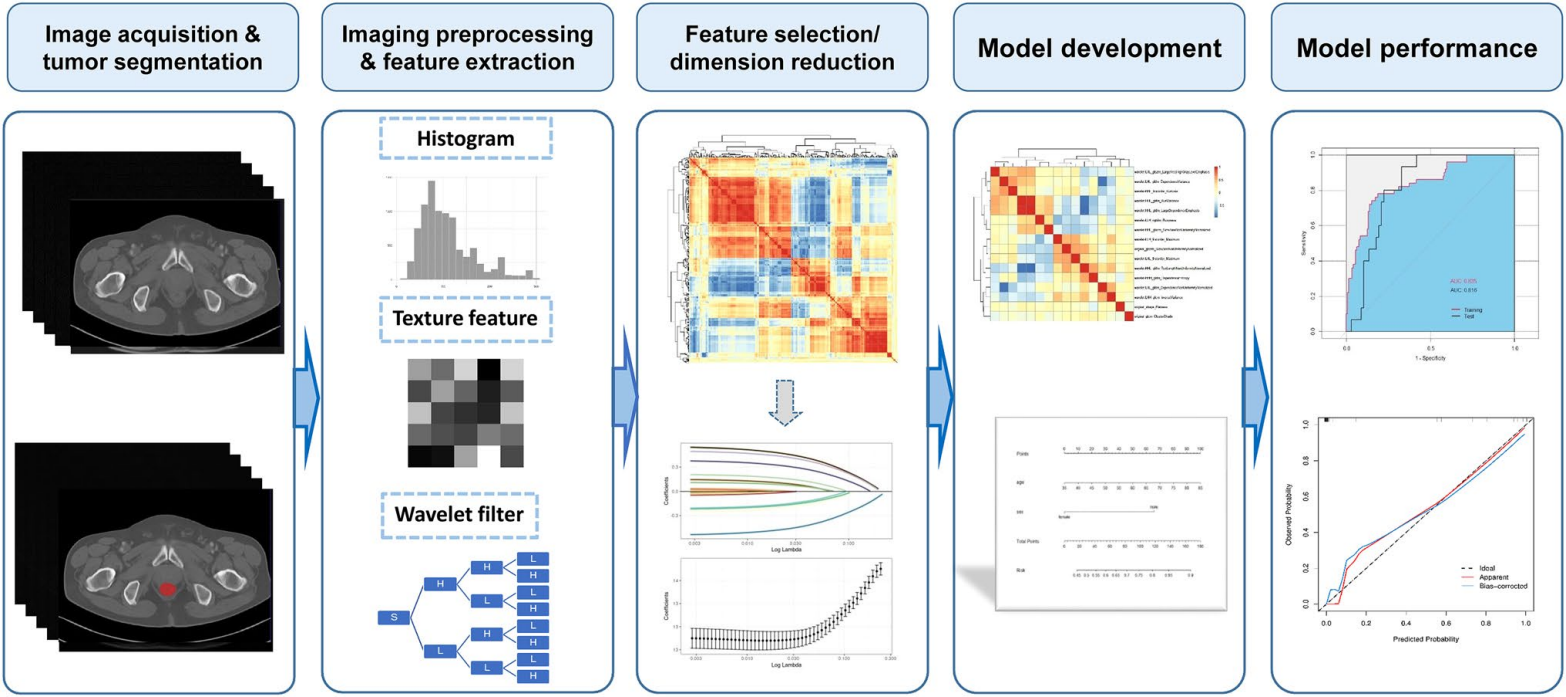
A radiomics study workflow

A predefined table was applied to extract the study information, which included: 1) basic study characteristics (for example the first author, publish year, country, and study design); 2) patient characteristics; 3) characteristics in radiomics workflow (such as tumor segmentation method, software used for radiomics feature extraction; a typical radiomics research workflow is shown in Fig. 1); 4) diagnostic performance metrics (true positives, false positives, false negatives, and true negatives) to construct a 2 × 2 table. When a study involved training and test cohorts, the diagnostic performance in the test cohort was selected for the model’s prediction power. If several prediction models were developed in one study, the model with the best performance was chosen. If the study did not have a test cohort, the predictive metrics in the validation cohort were extracted. When the provided data on diagnostic performance were insufficient to create a 2 × 2 table, an email was sent to the corresponding author for the missing information. The metrics were visualized as a forest plot to intuitively evaluate the predictive performance of the radiomics prediction models, which was achieved by using the software Review Manager (RevMan, version 5.3. Copenhagen: The Nordic Cochrane Centre, The Cochrane Collaboration, 2014).

The terms “validation” and “test” were unified in this study to avoid any confusion: “validation cohort” was defined as the part of the training cohort which was randomly divided for fine-tuning of super-parameters during modeling, while “test cohort” was defined as a hold-out dataset that was externally separate from the training cohort, not involved in the modeling [24]. The test cohort could be temporally or geographically independent from the training cohort [25]. “External cohort” and “test cohort” will be used interchangeably in this study.

### Assessment of radiomics quality score and the risk of bias

The tool used for methodological quality evaluation of the radiomics studies was the radiomics quality score (RQS) scale, which was proposed by Lambin and colleagues in 2017 [20]. The RQS scale consists of 16 items evaluating the research and reporting quality in the workflow of the radiomics model development. Different points are assigned to each item according to the degree the research achieves. The total points for this scoring system are 36, corresponding to 100% in percentage [20].

Research quality was also evaluated by using the Quality Assessment of Diagnostic Accuracy Studies 2 (QUADAS-2) criterion [26]. This tool assesses the risk of bias in a study in four dimensions: patient selection, index test, reference standard, and flow and timing, with results marked as low, high, and unclear risk indicating different levels of risk in each domain [26].

Data extraction and study quality evaluation were performed and cross-validated by the same two researchers ('Q.W' and 'J.X',). In case of a discrepancy occurring, the senior researcher ('T.B.B',) was consulted to reach an agreement.

## Results

**Fig. 2:**
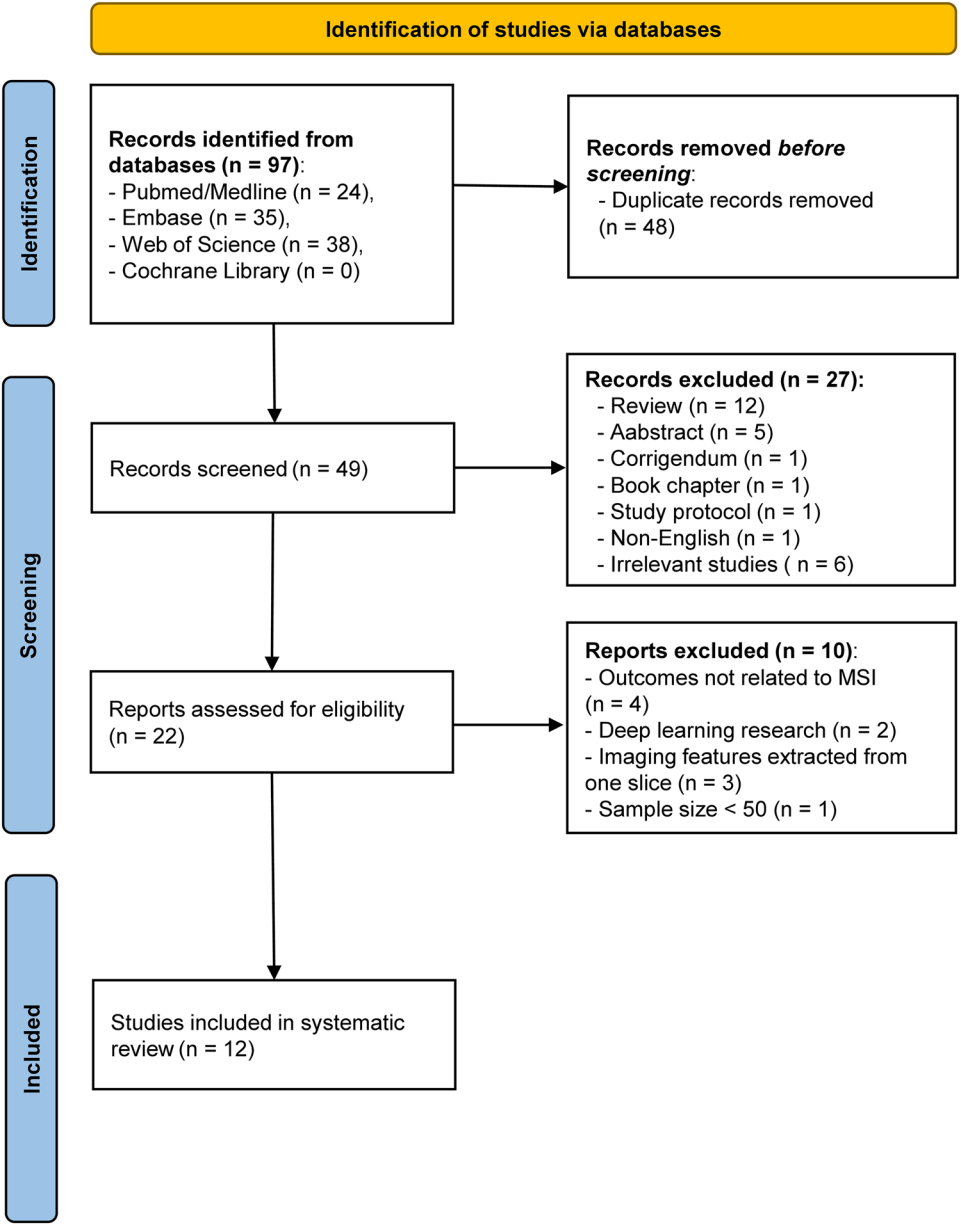
PRISMA flowchart of study selection

The initial search yielded 97 records from the four public databases. After the removal of 48 duplicates, 37 ineligible studies, 12 studies were finally included in this systematic review [27–38]. Among them, 10 studies with available data were able to construct a 2 × 2 contingency table [27–31, 33–35, 37, 38]. Figure 2 describes a PRISMA flowchart of the study selection.

### General characteristics and the incidence of MSI

The included studies were published between December 2019 and August 2022, and all studies were retrospectively designed (one study claimed to be prospective, but was judged as retrospective after discussion [32]). A total of 4,320 patients were included, with a sample size ranging from 90 to 837 (median 238) and a male/female ratio of 1.5 (2,592/1,728). Four studies were performed as multicenter research, with a sample size in the external cohorts ranging from 61 to 441 (median 82) [30, 35, 37, 38]. Five studies exclusively focused on rectal cancer, while the others on CRC [29, 32, 36–38].

Based on the surgically resected specimens, eleven studies evaluated the MSI status using the immunohistochemistry approach and one using the polymerase chain reaction method [31]. The incidence of MSI ranged from 8 to 34% (median: 19%). Among nine studies with available data, a majority of studies (8/9) reported an interval between imaging examination and surgery of less than 2 weeks [27, 29, 30, 33, 34, 36–38]. Table 1 provides detailed information about the basic characteristics of the included studies.

**Table 1:** Study and patient characteristics

Study ID	Year	Study design	Study center	Consecutive inclusion	Sample size (total)	Sample size (external cohort)	Age (Mean/ median)	Gender (M/F)	Indication	MSI criteria	MSI incidence	Interval between imaging and surgery
Fan et al. [27]	2019	R	Single	Yes	119	NA	60	79/40	Stage II CRC	IHC	25%	< 2 weeks
Pernicka et al. [28]	2019	R	Single	Yes	198	NA	52/62^#^	100/98	Stage II-III CRC	IHC	32%	8 weeks
Zhang et al.[29]	2021	R	Single	Yes	491	NA	61	318/173	RC	IHC	10%	2 weeks
Cao et al. [30]	2021	R	Two	Yes	502	61	59/57^†^	293/209	CRC	IHC	15%	2 weeks
Pei et al. [31]	2021	R	Single	Yes	762	NA	57	439/323	CRC	PCR	17%	Unclear
Zo.Li et al.[32]	2021	R	Single	Unclear	90	NA	61/58^#^	53/37	RC	IHC	33%	Unclear
J.Li et al.[33]	2021	R	Single	Yes	173	NA	61	99/74	CRC	IHC	8%	1 week
Ying et al.[34]	2022	R	Single	Yes	276	NA	64	154/122	CRC	IHC	19%	2 weeks
Chen et al.[35]	2022	R	Two	Yes	837	441	56–65	513/324	CRC	IHC	12%	Unclear
Yuan et al.[36]	2022	R	Single	Unclear	497	NA	63/64^#^	316/181	RC	IHC	19%	2 weeks
Jing et al.[37]	2022	R	Two	Unclear	176	65	57/59^†^	115/61	RC	IHC	18%/17%	2 weeks
Z.Li et al.[38]	2022	R	Three	Unclear	199	99	57	113/86	RC	IHC	34%	1 week

*Note *#in microsatellite stability and MSI groups, respectively; †in the training and test cohorts, respectively. *CRC*, colorectal cancer; *IHC*, immunohistochemistry; *MSI*, microsatellite instability; *NA*, not available/applicable; *PCR*, polymerase chain reaction; *R*, retrospective; *RC*, rectal cancer

### RQS and QUADAS-2 assessment

**Fig. 3:**
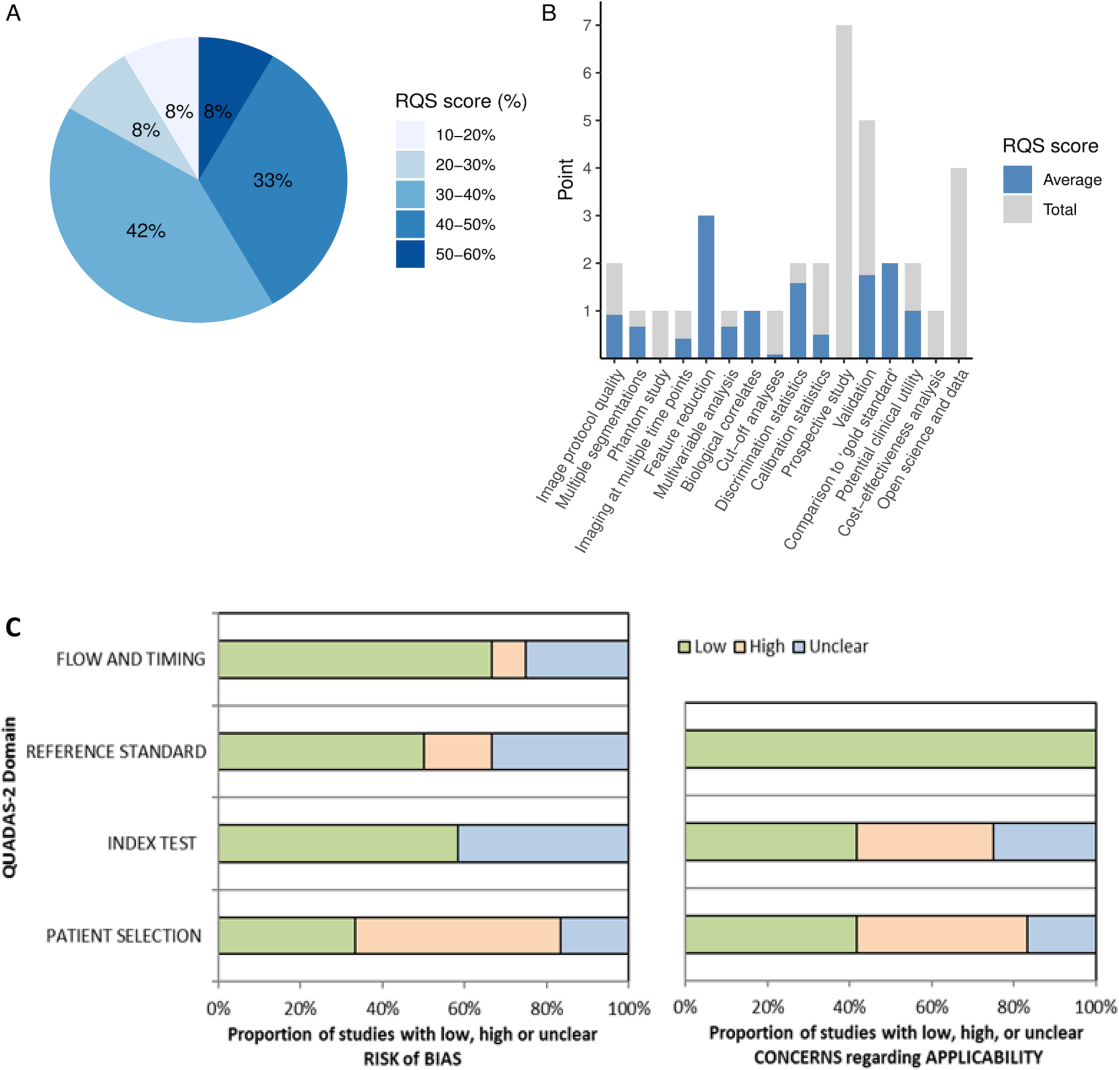
Methodological quality assessment of the radiomics studies by the radiomics quality score (A, B) and the quality assessment of the diagnostic accuracy studies (QUADAS-2) (C)

The median RQS score of the included studies was 13.5 points (range 5–18), corresponding to 38% (range 14–50%) of the full RQS score. The highest score of 50% was obtained in only one study [30]. The lowest score of 5 points (14%) was observed in an early study on this topic, and the main points were lost due to a lack of validation cohort [27]. Regarding performance in each item of the RQS, three items were fulfilled by all studies (100%): “feature reduction or adjustment,” “biological correlates,” and “comparison to gold standard.” On the other hand, four items (“phantom study,” “prospective study,” “cost-effectiveness analysis” and “open science and data”) were assigned 0 as none of the included studies involved them. A summary of the RQS score is presented in Fig. 3 A and B, and detailed information on the RQS score for each study is provided in Supplementary file 3.

A majority of the studies showed a low or unclear risk of bias and applicability concerns as evaluated by QUADAS-2 (Fig. 3 C). The main source of the high risk of bias and application concern was the domain of “patient selection” due to the retrospective nature of the studies, and patient selection bias seemed inevitable. Detailed evaluation of the included studies in each domain is provided in Supplementary file 4.

### Study characteristics

The study characteristics are described according to the five phases of a radiomics research workflow (Table 2):

**Table 2:** Characteristics of the radiomics study workflow

Study ID	Imaging modality	Phases/sequences	Segmentation manner	Imaging preprocessing	Feature extraction software	No. of imaging feature	Feature selection strategy	No. of Imaging features in model
Fan et al. [27]	CT	PVP	Semi-automatic	No	MATLAB	398	LASSO-logistic	6
Pernicka et al. [28]	CT	PVP	Manually	No	MATLAB	254	Wilcoxon rank sum; Correlation analysis	40
Zhang et al. [29]	MRI	T2WI	Manually	No	Pyradiomics	1454	ICC, t test, LASSO	6
Cao et al. [30]	CT	AP, PVP&DP	Manually	1 × 1 × 1&gray-level discretization	Pyradiomics	1037	ICC, Univariate analysis, LASSO, Multivariable logistic regression	16
Pei et al. [31]	CT	UP, PVP	Manually	Yes	MaZda	340	LASSO-logistic	16
Zo.Li et al. [32]	MRI	T2WI, ADC	Manually	No	AK software	385	RF, Multivariate logistic	4
J.Li et al. [33]	PET/CT	NA	Manually	Yes	Pyradiomics	2492	RF, Ensemble paradigm, Relevancy-based analysis, Non-redundancy-based analysis	2
Ying et al. [34]	CT	PVP	Manually	No	Artificial Intelligent Kit	1037	ICC, mRMR, LASSO	12
Chen et al. [35]	CT	PVP	Manually	1 × 1 × 1 &Hounsfield unit bin width discretization)	Pyradiomics	1037	*t* test/Mann–Whitney U test, RFE-SVM	10
Yuan et al. [36]	CT	PVP	Manually	1 × 1 × 1 & 1–32 gray-scale	AK software	792	ICC, variance, correlation analysis, LASSO	51
Jing et al. [37]	MRI	CE-T1WI, T2WI & DWI	Manually	Normalization	Radcloud	1409	ICC, variance threshold, select-k-best, LASSO	4
Z.Li et al. [38]	MRI	T1WI,CE-T1WI, T2WI&DWI	Manually	Normalization, gray-level discretization bin width of 5	Pyradiomics	6420	ICC, correlation analysis	20

Study ID	Balanced technique	Classifier	Cross validation	AUC (training)	AUC (validation)	Model form	Calibration curve	Decision curve analysis	Clinical variables in model
Fan et al. [27]	SMOTE	Bayes	Tenfold	0.75	No	NA	No	No	No
Pernicka et al. [28]	No	Random forest	No	0.80	0.79	NA	No	No	No
Zhang et al. [29]	No	XGBoost	Tenfold	0.99	0.90	NA	No	No	Gender, Age, MR-T stage, CEA, CA19-9
Cao et al. [30]	SMOTE	Logistic regression	Fivefold	0.90	0.96^#^	Nomogram	Yes	Yes	Age, Location, CEA
Pei et al. [31]	No	Logistic regression	No	0.79	0.77	Nomogram	Yes	Yes	Age, Location, Platelet, High density lipid
Zo.Li et al. [32]	SMOTE	Logistic regression	No	0.91	0.93	Formula	Yes	Yes	No
J.Li et al. [33]	Undersampling	Balance bagging, adaboost	No	No	0.83	NA	No	No	No
Ying et al. [34]	No	Logistic regression	Tenfold	0.87	0.90	Nomogram	Yes	Yes	Location, WBC,CT reported IFS, grade
Chen et al. [35]	Random under-/upsampling	Neural network	No	0.79	0.78^#^	NA	No	No	Age, location
Yuan et al. [36]	No	Logistic regression	No	0.84	0.74	Nomogram	Yes	No	Lymph node ratio, CEA & drinking
Jing et al. [37]	SMOTE	Logistic regression	Fivefold	0.91	0.87^#^	Nomogram	No	Yes	No
Z.Li et al. [38]	No	Random forest	Tenfold	0.78	0.78^#^	No formula	No	No	No

*Note* #the test cohort. *ADC*, apparent diffusion coefficient image; *AP*, arterial phase; *AUC*, area under the receiver operating characteristic curve; *DP*, delayed phase; *CA*19-9, carbohydrate antigen 19–9; *CEA*, carcinoembryonic antigen; *CT*, computed tomography, *ICC*, intra-class correlation; *IFS*, inflammatory response; *LASSO*, least absolute shrinkage and selection operator; *MRI*, magnetic resonance imaging; mRMR, minimal redundancy maximal relevance; MR-T stage, T stage-based MRI examination; *PET/CT*, positron emission tomography–computed tomography; *PVP*, portal vein phase; T2WI, T2-weighted image; *NA*, not available; *RFE*, recursive feature elimination; *SMOTE*, synthetic minority oversampling technique. *SVM*, support vector machine; *UP*, unenhanced phase; *WBC*, white blood cell; XGboost, eXtreme gradient boosting

**Fig. 4:**
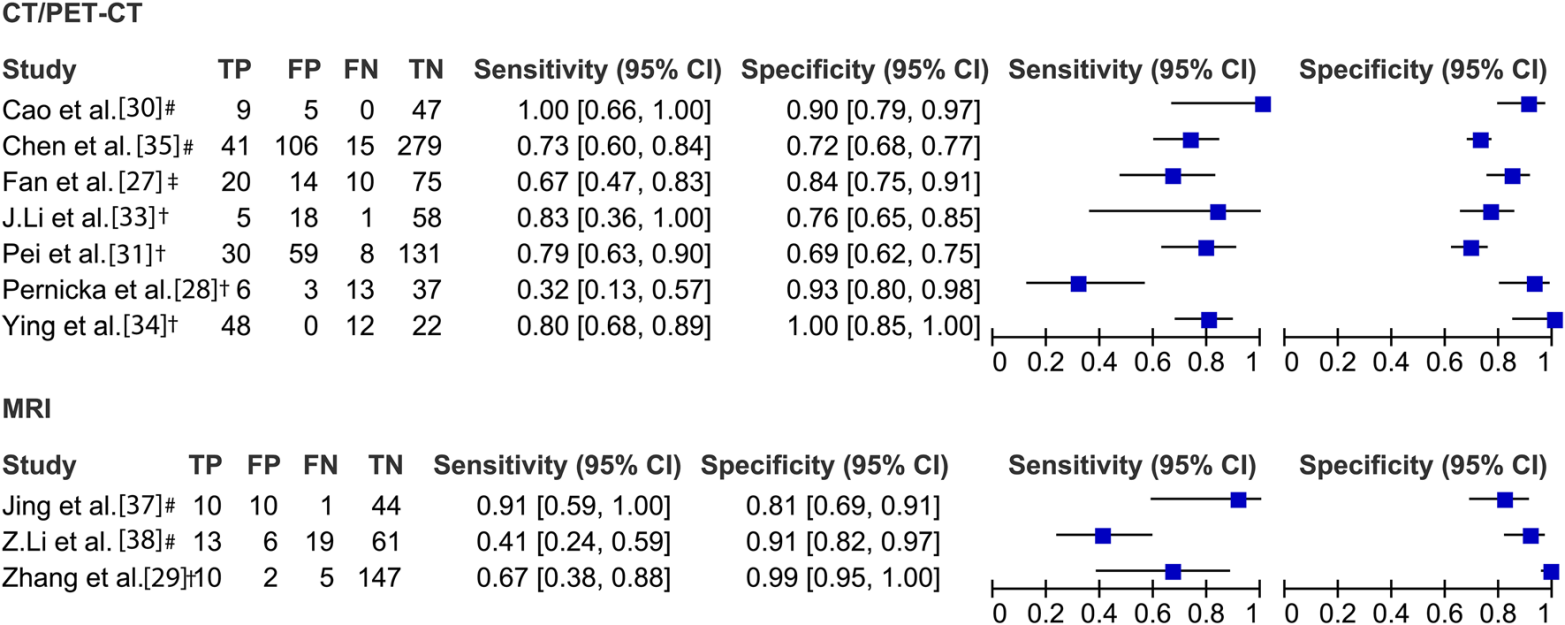
Performance metrics and forest plot of the sensitivity and specificity of the radiomics models in the prediction of microsatellite instability in patients with colorectal cancer. CI, confidence interval; CT, computed tomography; FN, false negative; FP, false positive; MRI, magnetic resonance imaging; PET/CT, positron emission tomography/CT; TN, true negative; TP, true positive. # data from the test cohort (i.e., the independent external cohort); † data from the validation cohort; ‡ data from the training cohort. Note that meta-analysis was not performed to synthesize the performance metrics due to the study heterogeneity

1. Imaging acquisition and tumor segmentation
  Among the included studies, seven used CT imaging, four MRI [29, 32, 37, 38], and one PET/CT [33]. Six studies applied images from one phase/sequence [27–29, 34–36]; the most frequently used phase was the portal venous phase of CT imaging (7/12) [27, 28, 30, 31, 34–36]. The tumor was segmented manually in 11 studies and semi-automatically in one [27].
2. Imaging preprocessing and feature extraction
  Seven studies stated imaging preprocessing before feature extraction [30, 31, 33, 35–38], but only five of them described their preprocessing techniques (resampling or gray-level discretization) [30, 35–38]. Pyradiomics was the most frequently used package for feature extraction (5/12) [29, 30, 33, 35, 38], and the number of the extracted radiomics features ranged from 254 to 6,420 (median: 1037).
3. Feature selection/dimension reduction
  All studies performed dimension reduction to select the most informative features and avoid potential model overfitting. The least absolute shrinkage and selection operator (LASSO) was the researchers’ favorite machine learning tool to reduce redundant features (7/12) [27, 29–31, 34, 36, 37], followed by correlation analysis (3/12) [28, 36, 38]. After feature selection, the number of radiomics features was reduced to 11 (range 2-51) to be included in the radiomics model.
  In six studies, inter-/intra-observer correlation coefficient analysis was not only used for the assessment of feature reproducibility and stability but also feature selection [29, 30, 34, 36–38].
4. Model development
  Due to the relatively low incidence of MSI, resampling techniques were applied to balance the negative/positive classifications in six studies [27, 30, 32, 33, 35, 37], among which the Synthetic Minority Oversampling Technique was the most frequently used algorithm (4/6) [27, 30, 32, 37]. Logistic regression was the most commonly used classifier for modeling (6/12) [30–32, 34, 36, 37]. Cross-validation with 5 or tenfold was applied in six studies (6/12) to avoid model overfitting and to determine the superparameter [27, 29, 30, 34, 37, 38]. Six studies evaluated the predictive value of clinicopathological variables [29–31, 34–36], in which tumor location and age (both 4/6) were the most frequent, significant indicators for the prediction of MSI status, followed by carcinoembryonic antigen (3/6). All those six studies then combined the studied variables with the calculated radiomics risk score into a compound clinical radiomics model to predict MSI status.
5. Model performance
  Four studies visualized their models as a nomogram [30, 31, 34, 36], two studies provided the formula [32, 37], and one study used radiomics-based artificial neural network [35]. The area under the receiver operator curve (AUC) of the prediction models ranged from 0.75 to 0.99 (median 0.84) in the training cohort, from 0.74 to 0.93 (median 0.83) in the validation cohort, and from 0.78 to 0.96 (median 0.83) in the test cohort [30, 35, 37, 38]. Among the 10 studies with available metrics data, the median sensitivity was 0.76 (range 0.32–1.00) and the median specificity was 0.87 (range 0.69–1.00) (Fig. 4). In specific, in the radiomics model based on CT or PET/CT, the median sensitivity was 0.79 (range 0.32–1.00) and the median specificity was 0.84 (range 0.69–1.00) [27, 28, 30, 31, 33–36]. Five studies evaluated the agreement between the model-predicted outcome and the observed outcome by plotting a calibration curve [30–32, 34, 36]. Decision curve analysis was performed among five studies to evaluate the clinical usefulness of their models [30–32, 34, 37].

## Discussion

This systematic review showed that radiomics models using the machine learning approach on pretreatment imaging modalities had a high predictive efficacy, with a median AUC of 0.83, a median sensitivity of 0.76, and a specificity of 0.87. Despite these promising results, the radiomics model is still far away from clinical utility due to the insufficient methodological quality as reflected by the low RQS score.

The translation of these prediction models into clinical routine settings is mainly determined by the study’s validity. Ideally, a reliable radiomics signature can be developed from a prospective, large sample cohort with a study population consecutively enrolled. Although none of the included studies was prospectively designed, the largest sample size was as high as 837 and almost half of the studies (5/12) had a sample size of over 490. The median incidence of MSI in the included studies was 19%, which was a little higher than the reported incidence (13–15%) [3, 39–42]. Two studies that did not state whether the subjects were consecutively included or not had an MSI incidence as high as 33% and 34% [32, 38]. That might be due to their case–control study design (1:2). In diagnostic test studies, this type of study design is prone to overestimate the performance of the prediction model and should be avoided as it cannot reflect the real-world situation [26]. One may argue that when performing machine learning algorithms, the positive and negative classifications of a cohort should be balanced to avoid potential overfitting. In fact, several techniques have been proposed to deal with this situation, such as the Synthetic Minority Oversampling Technique [43, 44]. Half of the reviewed studies adopted techniques to cope with the imbalanced classifications [28, 32, 35, 36, 38].

Before translating the radiomics models into clinical implementation, it is also vital to verify the model in an external cohort [25]. Given that the model developed in the training cohort tends to be overfitting, the external cohort can be used to evaluate the generalization of a prediction model and provide a real performance of the model in real-world practice [45]. One-third of the studies (4/12) tested their models in an external cohort, yielding a median AUC of 0.83 [32, 34, 38]. On the other hand, internal validation using cross-validation or bootstrapping techniques within the training cohort plays an equivalent role to avoid potential overfitting and to optimize the prediction model [45-47]. Six studies adopted five-/tenfold cross-validation when developing their models.

Researchers should also make their prediction model reproducible and validated by other investigators. The first step could be to deposit the radiomics codes/data at a public platform (such as https://github.com) or to provide more details on software usage. However, none of the included research published their code or data, resulting in a zero score for the “open science and data” item in the RQS scale. Besides, the models should also be presented in a proper and easy-to-use form for clinical usage, for example, present as a nomogram. Six studies provided the formula and/or nomogram, which forwarded one step for their models validated by other centers. Furthermore, the determination of the optimal cutoff value of the prediction model is often a trade-off between sensitivity and specificity. Its important role has been emphasized by a specific item in the RQS score. The knowledge of the specific cutoff value of a model makes it possible for other researchers to validate the model. However, only two studies stated the cutoff values of their models [32, 35]. When implementing the prediction model, researchers should also be aware of the target patient population or subpopulation. The patients in the included studies had different indications, where some studies merely focused on rectal cancer or CRC stage II/III, while others were on the general CRC population.

RQS is a commonly used tool for the appraisal of radiomics research quality [20]. As it evaluates the key steps in the radiomics research workflow, RQS has the potential to become not only a guide when performing the radiomics study, but also a useful checklist when submitting their manuscript to a journal. The included studies fulfilled well in three domains of the RQS scale, accounting for 17% of the full score (6 points). Besides, more than half of the studies (7/12) reported both a discriminative performance and a resampling technique in the item of “discrimination statistics”, earning an average of 1.6 points for this item. However, the included studies in this review only yielded a median score of 13.5 points (corresponding to 38% of the full score of 36) and the highest score of 18 points (50% of the full score). The main reason was that four domains in the RQS scale were not in response by any of the included studies, for example, to make their code/data public. These four domains account for 39% of the full scale (14 points). However, the RQS scale may assign a too-high weight to the item “prospective study” (7 points), which is approximately equal to 20% of the full score. This is a relatively high score given that most other items in the RQS tool often have a maximum of 1–2 point(s). However, no prospective studies were included in this systematic review, which further contributed to a lower RQS score in the included studies.

On the other hand, other appraisal tools, such as QUADAS-2, which was designed for the appraisal of the general diagnostic test studies, should also be adopted to complement the RQS tool in the assessment of radiomics research quality. For instance, the RQS scale does not involve patient selection, but this issue is of clinical importance when evaluating a diagnostic test study. In the QUADAS-2, patient selection is one of the four main constituent dimensions. Besides, other commonly used guidelines, such as the “checklist for artificial intelligence in medical imaging” (CLAIM) [48] and the “transparent reporting of a multivariable prediction model for individual prognosis or diagnosis (TRIPOD)” statement [25], may also be beneficial to conduct a rigorous and reproducible radiomics study and to improve the research and reporting quality.

There are some limitations in this study. First, the number of included studies was relatively limited, no study was prospectively designed, and only four studies validated their models in external cohorts. These limitations may undermine the conclusion drawn from our study. On the other hand, the limited number of studies, as shown by the initial records retrieved from the four databases, also reflects that this topic (using radiomics approach for predicting gene expression levels in CRC) is relatively novel and the research is still at its early stage. Second, the included studies were heterogeneous not only in the imaging modalities and phase/sequence used but also in the imaging features and modeling strategies. In this context, a meta-analysis to synthesize the diagnostic metrics was not performed and a pooled AUC for the radiomics model in the prediction of MSI status was therefore absent. Third, deep learning studies were not included due to the poor interpretability of deep learning-derived imaging features. This is also a burgeoning field where the deep learning model is often assumed to have higher accuracy than the radiomics models [49]. Lastly, although RQS is a useful tool in the assessment of radiomics research quality, it has limitations. Further revision of RQS might make it more comprehensive in the quality appraisal of the radiomics studies.

## Conclusions

In conclusion, despite radiomics models derived from pretreatment imaging modalities having a high performance in the prediction of MSI status in CRC patients, radiomics does not seem to be ready to serve as an imaging biomarker utilized in clinical practice due to the insufficient methodological quality of the research.

## Supplementary Information

Below is the link to the electronic supplementary material.

Supplementary file1 (DOC 66 KB)

Supplementary file2 (DOCX 20 KB)

Supplementary file3 (DOCX 16 KB)

Supplementary file4 (DOCX 15 KB)

## Data Availability

The datasets supporting the conclusions of this article are included within the article and its supplementary files.

## Abbreviations

AUC: Area under the receiver operator curve
CI: Confidence interval
CRC: Colorectal cancer
CT: Computed tomography
MRI: Magnetic resonance imaging
MSI: Microsatellite instability
PET/CT: Positron emission tomography/CT
QUADAS-2: Quality assessment of diagnostic accuracy studies 2
RQS: Radiomics quality score
