# Supplementary material for: Systematic review of machine learning-based radiomics approach for predicting microsatellite instability status in colorectal cancer
Source: Radiol Med. 2023 Jan 17;128(2):136–48. doi: 10.1007/s11547-023-01593-x (PMC9938810; doi:10.1007/s11547-023-01593-x)
Supplement: Supplementary file 2 — Supplementary file2 (DOCX 20 KB) [file 11547_2023_1593_MOESM2_ESM.docx]

Table s1:

Literature searching strategies in PubMed, Web of science, Embase and Cochrane Library (updated on 10 November, 2022).

| **No.** | **Search Query for PubMed** | **Result** |
| --- | --- | --- |
| #1 | "colorectal neoplasms"[MeSH Terms] OR "colonic neoplasms"[MeSH Terms] OR "rectal neoplasms"[MeSH Terms] | 230,207 |
| #2 | "colorectal neoplasms"[Title/Abstract] OR "colorectal metastases"[Title/Abstract] OR "colorectal cancer"[Title/Abstract] OR "CRC"[Title/Abstract] OR "colonic neoplasms"[Title/Abstract] OR "colon cancer"[Title/Abstract] OR "rectal neoplasms"[Title/Abstract] OR "rectal cancer"[Title/Abstract] OR "colorectal cancer liver metastases"[Title/Abstract] OR "colorectal liver metastases"[Title/Abstract] OR "CRLM"[Title/Abstract] | 196,935 |
| #3 | **#1 OR #2** | 287,955 |
| #4 | "microsatellite instability"[MeSH Terms] | 4,434 |
| #5 | ‘microsatellite instability’[Title/Abstract] OR ‘MSI’[Title/Abstract] OR ‘MMR’[Title/Abstract] OR ‘mismatch repair’[Title/Abstract] OR ‘MMRd’[Title/Abstract] OR ‘dMMR’ [Title/Abstract] | 28,864 |
| #6 | **#4 OR #5** | 29,420 |
| #7 | "radiogenomi*"[Title/Abstract] OR "imaging genomic"[Title/Abstract] OR "imaging genomics"[Title/Abstract] OR "texture"[Title/Abstract] OR "textural"[Title/Abstract] OR "radiomi*"[Title/Abstract] OR "imaging features"[Title/Abstract] OR "imaging biomarker"[Title/Abstract] OR "imaging biomarkers"[Title/Abstract] OR "imaging characteristics"[Title/Abstract] | 72,818 |
| #8 | **#3 AND #6 AND #7** | 24 |

| **No.** | **Search Query for Web of Science** | **Result** |
| --- | --- | --- |
| #1 | TS = ("colorectal neoplasms" OR "colorectal metastases" OR "colorectal cancer" OR "CRC" OR "colonic neoplasms" OR "colon cancer" OR "rectal neoplasms" OR "rectal cancer" OR "colorectal cancer liver metastases" OR "colorectal liver metastases" OR "CRLM") | 291,024 |
| #3 | TS = (‘microsatellite instability’ OR ‘MSI’ OR ‘MMR’ OR ‘mismatch repair’ OR ‘MMRd’ OR ‘dMMR’) | 47,468 |
| #4 | TS = ("radiogenomi*" OR "imaging genomic" OR "imaging genomics" OR "texture" OR "textural" OR "radiomi*" OR "imaging features" OR "imaging biomarker" OR "imaging biomarkers" OR "imaging characteristics") | 239,015 |
| #5 | **#1 AND #2 AND #3** | 38 |

| **No.** | **Search Query for Embase** | **Result** |
| --- | --- | --- |
| #1 | "colorectal neoplasms"/exp OR "colonic neoplasms"/exp OR "rectal neoplasms"/exp | 449, 027 |
| #2 | "colorectal neoplasms":ab,ti OR "colorectal metastases":ab,ti OR "colorectal cancer":ab,ti OR "CRC":ab,ti OR "colonic neoplasms":ab,ti OR "colon cancer":ab,ti OR "rectal neoplasms":ab,ti OR "rectal cancer":ab,ti OR "colorectal cancer liver metastases":ab,ti OR "colorectal liver metastases":ab,ti OR "CRLM":ab,ti | 288,381 |
| #3 | **#1 OR #2** | 478,461 |
| #4 | "microsatellite instability"/exp | 18,546 |
| #5 | ‘microsatellite instability’:ab,ti OR ‘MSI’:ab,ti OR ‘MMR’:ab,ti OR ‘mismatch repair’:ab,ti OR ‘MMRd’:ab,ti OR ‘dMMR’:ab,ti | 43,772 |
| #6 | **#4 OR #5** | 49,128 |
| #7 | "radiogenomi*":ab,ti OR "imaging genomic":ab,ti OR "imaging genomics":ab,ti OR "texture":ab,ti OR "textural":ab,ti OR "radiomi*":ab,ti OR "imaging features":ab,ti OR "imaging biomarker":ab,ti OR "imaging biomarkers":ab,ti OR "imaging characteristics":ab,ti | 91,930 |
| #8 | **#3 AND #6 AND #7** | 35 |

| **No.** | **Search Query for Cochrane Library** | **Result** |
| --- | --- | --- |
| #1 | MeSH descriptor: [Colorectal Neoplasms] explode all trees OR MeSH descriptor: [Colonic Neoplasms] explode all trees OR MeSH descriptor: [Rectal Neoplasms] explode all trees | 9,341 |
| #2 | "colorectal neoplasms":ti,ab,kw OR "colorectal metastases":ti,ab,kw OR "colorectal cancer":ti,ab,kw OR CRC:ti,ab,kw OR "colonic neoplasms":ti,ab,kw OR "colon cancer":ti,ab,kw OR "rectal neoplasms":ti,ab,kw OR "rectal cancer":ti,ab,kw OR "colorectal cancer liver metastases":ti,ab,kw OR "colorectal liver metastases":ti,ab,kw OR CRLM:ti,ab,kw | 23,081 |
| #3 | **#1 OR #2** | 23,264 |
| #4 | Mesh descriptor [microsatellite instability] explode all trees | 60 |
| #5 | "microsatellite instability":ti,ab,kw OR MSI:ti,ab,kw OR MMR:ti,ab,kw OR "mismatch repair":ti,ab,kw OR MMRd:ti,ab,kw OR dMMR:ti,ab,kw | 1,729 |
| #6 | **#4 OR #5** | 1,729 |
| #7 | "radiogenomi*":ti,ab,kw OR "imaging genomic":ti,ab,kw OR "imaging genomics":ti,ab,kw OR "texture":ti,ab,kw OR "textural":ti,ab,kw OR "radiomi*":ti,ab,kw OR "imaging features":ti,ab,kw OR "imaging biomarker":ti,ab,kw OR "imaging biomarkers":ti,ab,kw OR "imaging characteristics":ti,ab,kw | 2,498 |
| #8 | **#3 AND #6 AND #7** | 0 |
