# Supplementary material for: Systematic review of machine learning-based radiomics approach for predicting microsatellite instability status in colorectal cancer
Source: Radiol Med. 2023 Jan 17;128(2):136–48. doi: 10.1007/s11547-023-01593-x (PMC9938810; doi:10.1007/s11547-023-01593-x)
Supplement: Supplementary file 3 — Supplementary file3 (DOCX 16 KB) [file 11547_2023_1593_MOESM3_ESM.docx]

Supplementary file 3. Research quality assessment by the Radiomics Quality Score tool

| Study ID | Image protocol quality | Multiple segmentations | Phantom study | Imaging at multiple time points | Feature reduction | Multivariable analysis with non radiomics features | Biological correlates | Cut-off analyses | Discrimination statistics | Calibration statistics | Prospective study | Validation | Comparison to ‘gold standard’ | Potential clinical utility | Cost-effectiveness analysis | Open science and data | Total points |
| --- | --- | --- | --- | --- | --- | --- | --- | --- | --- | --- | --- | --- | --- | --- | --- | --- | --- |
| Fan et al.[27] | 1 | 0 | 0 | 0 | 3 | 1 | 1 | 0 | 2 | 0 | 0 | -5 | 2 | 0 | 0 | 0 | 5  (14%) |
| Pernicka et al.[28] | 1 | 0 | 0 | 0 | 3 | 1 | 1 | 0 | 1 | 0 | 0 | 2 | 2 | 0 | 0 | 0 | 11 (31%) |
| Zhang et al.[29] | 1 | 1 | 0 | 0 | 3 | 1 | 1 | 0 | 2 | 0 | 0 | 2 | 2 | 0 | 0 | 0 | 13 (36%) |
| Cao et al.[30] | 1 | 1 | 0 | 1 | 3 | 1 | 1 | 0 | 2 | 1 | 0 | 3 | 2 | 2 | 0 | 0 | 18 (50%) |
| Pei et al.[31] | 0 | 1 | 0 | 1 | 3 | 1 | 1 | 0 | 1 | 1 | 0 | 2 | 2 | 2 | 0 | 0 | 15 (42%) |
| Zo.Li et al.[32] | 1 | 0 | 0 | 1 | 3 | 0 | 1 | 0 | 1 | 1 | 0 | 2 | 2 | 2 | 0 | 0 | 14 (39%) |
| J.Li et al.[33] | 1 | 0 | 0 | 0 | 3 | 0 | 1 | 0 | 1 | 0 | 0 | 2 | 2 | 0 | 0 | 0 | 10 (28%) |
| Ying et al.[34] | 1 | 1 | 0 | 0 | 3 | 1 | 1 | 0 | 2 | 1 | 0 | 2 | 2 | 2 | 0 | 0 | 16 (44%) |
| Chen et al.[35] | 1 | 1 | 0 | 0 | 3 | 1 | 1 | 1 | 2 | 0 | 0 | 3 | 2 | 2 | 0 | 0 | 17 (47%) |
| Yuan et al.[36] | 1 | 1 | 0 | 0 | 3 | 1 | 1 | 0 | 1 | 1 | 0 | 2 | 2 | 0 | 0 | 0 | 12 (33%) |
| Jing et al.[37] | 1 | 1 | 0 | 1 | 3 | 0 | 1 | 0 | 2 | 1 | 0 | 3 | 2 | 2 | 0 | 0 | 16 (44%) |
| Z.Li et al. [38] | 1 | 1 | 0 | 1 | 3 | 0 | 1 | 0 | 2 | 0 | 0 | 3 | 2 | 0 | 0 | 0 | 13 (36%) |
