# Supplementary material for: Systematic review of machine learning-based radiomics approach for predicting microsatellite instability status in colorectal cancer
Source: Radiol Med. 2023 Jan 17;128(2):136–48. doi: 10.1007/s11547-023-01593-x (PMC9938810; doi:10.1007/s11547-023-01593-x)
Supplement: Supplementary file 4 — Supplementary file4 (DOCX 15 KB) [file 11547_2023_1593_MOESM4_ESM.docx]

Supplementary file 4. Research quality assessment by the Quality Assessment of Diagnostic Accuracy Studies 2 tool.

| Study ID | Risk of bias | | | | Application concerns | | |
| --- | --- | --- | --- | --- | --- | --- | --- |
|  | Patient selection | Index  test | Reference  standard | Flow and timing | Patient selection | Index  test | Reference  standard |
| Fan et al.[27] | Low | Unclear | Unclear | Low | Unclear | High | Low |
| Pernicka et al.[28] | High | Low | Low | High | Unclear | High | Low |
| Zhang et al.[29] | Low | Low | Low | Low | High | Unclear | Low |
| Cao et al.[30] | Low | Unclear | Unclear | Low | Low | Low | Low |
| Pei et al.[31] | Low | Low | Low | Unclear | Low | Low | Low |
| Zo.Li et al.[32] | High | Unclear | Low | Unclear | High | Unclear | Low |
| J.Li et al.[33] | Unclear | Low | Low | Low | Low | High | Low |
| Ying et al.[34] | High | Low | Unclear | Low | Low | Low | Low |
| Chen et al.[35] | High | Low | Unclear | Unclear | Low | Low | Low |
| Yuan et al.[36] | Unclear | Unclear | High | Low | High | High | Low |
| Jing et al.[37] | High | Unclear | High | Low | High | Low | Low |
| Z.Li et al.[38] | High | Low | Low | Low | High | Unclear | Low |
